# Intensified Antituberculosis Therapy Regimen Containing Higher Dose Rifampin for Tuberculous Meningitis: A Systematic Review and Meta-Analysis

**DOI:** 10.3389/fmed.2022.822201

**Published:** 2022-02-25

**Authors:** Mengmeng Zhang, Minggui Wang, Jian-Qing He

**Affiliations:** Department of Respiratory and Critical Care Medicine, West China Hospital, Sichuan University, Chengdu, China

**Keywords:** tuberculous meningitis, high-dose rifampin, pharmacokinetics parameters, survival, meta-analysis

## Abstract

**Background:**

Tuberculous meningitis is difficult to diagnose and is associated with high mortality. Recently, several studies evaluated the intensified regimen containing higher dose rifampin to treat tuberculous meningitis. However, this topic remains to be concluded. Therefore, this systematic review and meta-analysis was conducted to evaluate pharmacokinetics parameters, safety, and survival benefits of high-dose rifampin for tuberculous meningitis.

**Method:**

Data were searched from PubMed, EMBASE, The Cochrane Library, and Web of Science for studies describing an antituberculosis regimen including a higher dose of rifampin for patients with tuberculous meningitis. The quality of eligible studies was evaluated *via* The Cochrane Risk of Bias Tool. The meta-analysis was performed by Review Manager 5.3 software, the synthesis of the data was shown in mean difference (MD) or relative risk (RR), and 95% confidence intervals (CIs).

**Results:**

There were six randomized control trails included in this meta-analysis. The results showed that the concentration in plasma and cerebrospinal fluid (CSF) were significantly higher in the intervention group than the standard group [MD = 22.08, 95%CI (16.24, 27.92), *p* < 0.00001; MD = 0.74, 95%CI (0.42, 1.05), *p* < 0.00001], as well as the area under the time concentration curve between 0 and 24 h (AUC_0−24_) of rifampin [MD 203.56, 95%CI (153.07, 254.05), *p* < 0.00001] in plasma, but the overall survival did not improve [RR = 0.92, 95%CI (0.67, 1.26), *p* = 0.61]. For adverse events, the results showed a statistically significant lower incidence of hypersensitivity compared with the intervention group [RR = 1.72, 95%CI (1.13, 2.62), *p* = 0.01]. Fortunately, other common adverse drug reactions such as liver injury, neurological events, myelosuppression, and cardiotoxicity had no significant increase [RR = 0.98, 95%CI (0.77, 1.26), *p* = 0.90; RR = 1.10, 95%CI (0.94, 1.30), *p* = 0.23; RR = 0.82, 95%CI (0.59, 1.13), *p* = 0.22; RR = 1.11, 95%CI (0.66, 1.86), *p* = 0.70].

**Conclusion:**

This meta-analysis suggested that the intensified treatment regimen including a higher dose of rifampin significantly increased the rifampin concentration both in the plasma and CSF, and it was safe in patients with tuberculous meningitis, but resulted in no improvement in survival rates.

## Introduction

Tuberculosis (TB) is a common disease caused by *Mycobacterium tuberculosis*. It typically affects the lungs which is called pulmonary tuberculosis, however, the disease can also affect other areas such as bones, lymph nodes, abdomen, central nervous system, and so on, named as extrapulmonary tuberculosis. According to the 2020 World Health Organization global tuberculosis report, approximately 10.0 million people fell ill with TB in 2019, and 1.4 million people died because of TB infection (1). As we can see, tuberculosis remains to be the world's most infectious disease. Tuberculous meningitis (TBM) is the most severe form of the disease, resulting from dissemination of *M. tuberculosis* to the cerebrospinal fluid (CSF) and meninges, accounting for approximately 5 to 10% of all extrapulmonary TB (2). TBM frequently occurs in children and immunocompromised patients, for example, HIV-infected individuals (3). The clinical features of TBM are non-specific and are often similar to the symptoms of other neurological conditions such as headache, fever, fatigue, loss of appetite, weight loss, nausea, vomiting, and so on, making early diagnosis difficult, and even with treatment, it can also cause high mortality as well as different sequela. A review from Seddon et al. reported that about 100,000 individuals develop TBM annually, but this figure may be much higher due to many cases of TBM remaining undiagnosed (3). In patients that have HIV and TBM coinfection, the mortality rate may be up to 50% (4).

On the basis of the fourth edition of the WHO Treatment of Tuberculosis Guidelines, drug-susceptible TBM treatment therapy is based on the regimens of pulmonary tuberculosis, consisting of four main drugs including isoniazid, rifampin, pyrazinamide, and ethambutol. Given the critical risk of mortality and disability, the guidelines recommend 9–12 months of treatment for TBM containing intensive phase and continuation phase treatment (5). Rifampin is the key sterilizing drug in tuberculosis treatment, the WHO treatment guidelines recommend a dose of rifampin of 8–12 mg/kg daily. However, rifampin has a high plasma protein binding rate, with about 70-80% rifampin protein-bound. This may cause a limiting free rifampin concentration in plasma and influence the treatment outcomes (6, 7). Compared to serum, it is likely that rifampin does not penetrate the blood–brain barrier well, so the CSF rifampin concentration scarcely exceeds the rifampin minimum inhibitory concentration (MIC) of *M. tuberculosis* (6). Ruslami et al. (8) performed a prospective observational pharmacokinetic study to assess the plasma and CSF concentration of three antituberculosis drugs in Indonesian children and adolescents. Finally, the results showed that the 20 participants had lower rifampin concentrations in CSF than in plasma and the rifampin concentration was extremely low in CSF. As noted above, the author suggested that there is a great request to increase the rifampin dose in children and adolescents diagnosed with TBM. As for adults, Mezochow et al. (9) demonstrated that when rifampin is administered in line with standard weight-based dosing guidelines, TBM patients are rarely expected to attain therapeutic rifampin exposure in CSF, and doubling the rifampin dose led to remarkable improvement in the probability of pharmacodynamic target attainment. In recent years, a series of clinical trials evaluated higher dose rifampin in a treatment regimen for TBM, however, the research findings appeared divided. In a 2013 study by Ruslami et al. (10), including 60 participants in Indonesia, the intervention group was given rifampin at a dose of 13 mg/kg/day (600 mg daily) intravenously for the first 14 days, alongside a different dose of moxifloxacin or not, meanwhile, the controlled group was administered 10 mg/kg/day (450 mg daily) of rifampin orally. The result showed that the rifampin plasma and CSF exposures in the intervention group were dramatically increased, approximately three times higher than the standard group. The author also found lower 6 months mortality in the high-dose rifampin group without increasing the risk for adverse events. But in a larger sample, randomized, double-blind, placebo-controlled trial containing 817 individuals conducted by Heemskerk et al. (11) in Vietnam, no survival benefit was observed when receiving a rifampin dose of 15 mg/kg/day as well as levofloxacin for the first 8 weeks, compared with the standard dose of 450 mg orally. Hence, as mentioned above, whether a rifampin dose more than 10 mg/kg/day would be beneficial to the treatment outcome of TBM is controversial at present.

Therefore, we conducted a meta-analysis of all relevant published studies to assess the pharmacokinetic parameters and clarify the efficacy and safety of the therapy regimens containing a higher dose of rifampin in patients with TBM.

## Methods

### Search Strategy and Selection Criteria

In this systematic review and meta-analysis, we searched the electronic databases PubMed, Embase, Web of Science, and The Cochrane Library for English language articles published up to 1 July, 2021, with the search terms (((“tubercul^*^” OR “TB”) AND “mening^*^”) OR “central nervous system tuberculosis”) AND “rifampi^*^”). The search strategy consisted of entry terms and MeSH terms. Studies were considered eligible based on the following criteria: (1) The original study population were people with a diagnosis of tuberculous meningitis. The diagnosis of tuberculous meningitis could be achieved by clinical diagnosis, microbiological confirmation, molecular tests such as nucleic acid amplification or Xpert MTB/RIF Ultra, brain imaging like computed tomography (CT) and magnetic resonance imaging (MRI), and immunodiagnostic tests such as interferon gamma release assays. (2) The intervention group received an intensified antituberculosis regimen consisting of a weight-based dose of rifampin more than 10 mg/kg/day; the delivery method could be intravenous or oral, and alongside other antituberculous drugs. (3) The control group accepted standard therapy containing rifampin at 10 mg/kg/day, along with other antituberculosis medications. (4) Articles described treatment outcomes including pharmacokinetic parameters for rifampin in serum containing an area under the time concentration curve between 0 and 24 h (AUC_0−24_), maximum concentration (C_max_) in plasma and CSF, mortality, and adverse events (AEs). A study was excluded if it was a case report, case series, comment, editorial, letter, review (including meta-analysis), animal experiment, or the full text was unavailable.

### Data Extraction

Two reviewers independently assessed the full text of the included studies and performed the data extraction. If there were discrepancies, a third investigator would make the definitive decision for the disagreement. The extracted data from included articles contained title, first author, published year, study period, the country where the study was performed, study design, follow-up period, sample size (standard dose rifampin group/higher dose rifampin group), HIV infection, glucocorticoids used or not, standard rifampin dosage, standard treatment regimen, high-dose rifampin dosage, intensified regimen, and treatment outcomes.

### Quality Assessment

The risk of bias of the included studies was assessed using the Cochrane Risk of Bias Tool, which was recommended by the Cochrane handbook version 5.10 (12). It includes the following seven aspects: (1) random sequence generation (selection bias); (2) allocation concealment (selection bias); (3) blinding of participants and personnel (performance bias); (4) blinding of outcome assessment (detection bias); (5) incomplete outcome data (attrition bias); (6) selective reporting (reporting bias); and (7) other bias. Each study was evaluated as “high risk”, “low risk”, or “unclear risk”. Two investigators independently analyzed each included study and finished the quality assessment. Any differences during the quality assessment were resolved by a third author.

### Statistical Analysis

The outcomes were analyzed by RevMan 5.3 software. The dichotomous variables were expressed as ratio and frequency, while the continuous variables were reported as mean and standard derivation (SD). For an included study containing the data of the median as well as the maximum and the minimum, the results were analyzed after the data were transformed through the conversion formula. The effect size of the intervention on dichotomous variables and continuous variables was shown in risk ratio (RR) with 95% confidence intervals (CIs) and mean difference (MD) with 95%CIs, respectively. Results were considered as significant when *P* < 0.05. The studies' heterogeneity was assessed by the Chi-square (α = 0.1) and I-square tests, with *P* < 0.1 and *I*^2^ > 50% indicating heterogeneity. A random effects model was applied regardless of heterogeneity. If heterogeneity was observed among the studies, subgroup analysis was performed to investigate the possible source of heterogeneity.

## Results

### Included Studies

**Figure 1:**
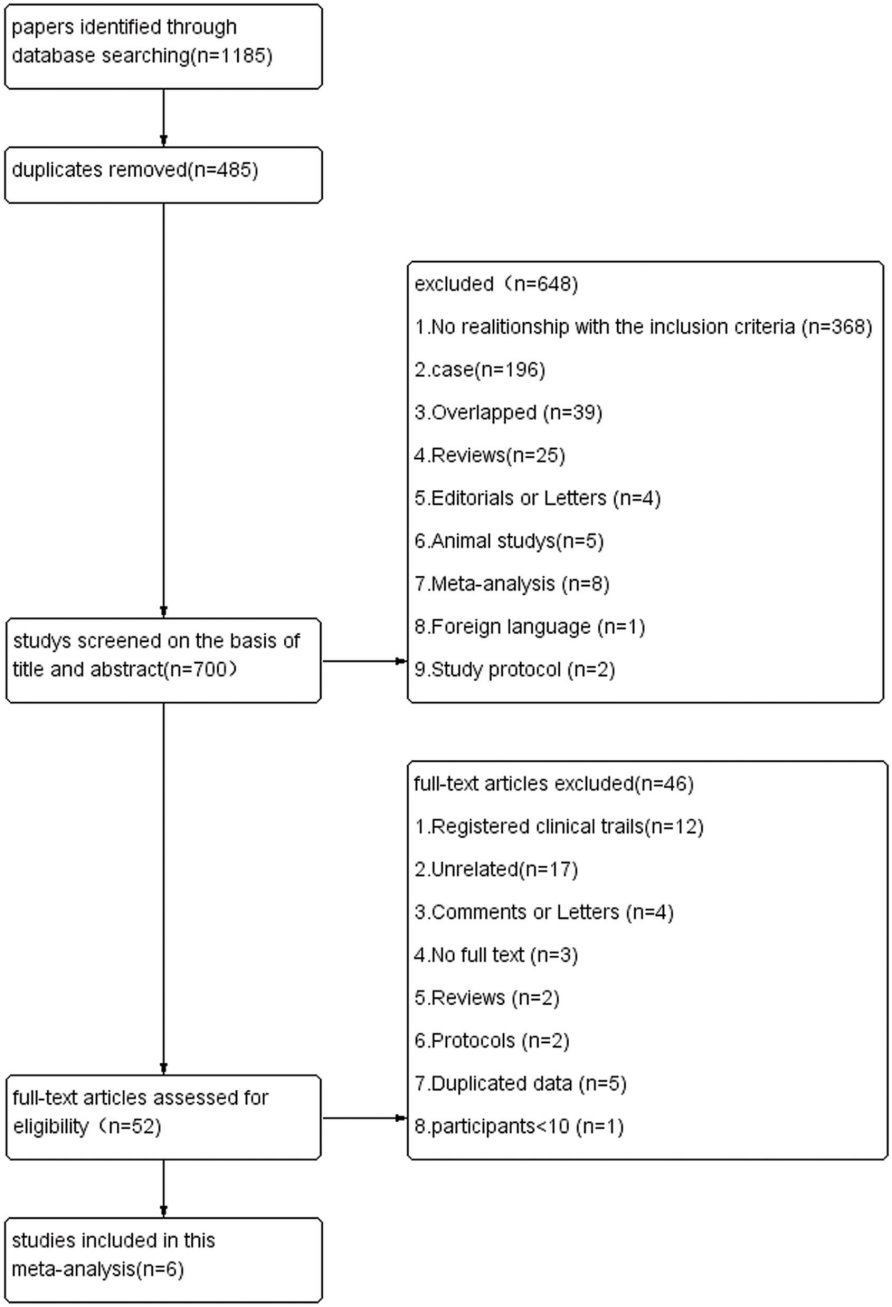
Flow diagram of studies.

A total of 1,185 published studies were retrieved from the electronic databases. After removing 485 duplicated articles, a total of 700 records remained. Then these studies were screened by the titles and abstracts, after this step, 648 studies were excluded. A total of 52 studies were selected for careful full-text assessment. Finally, six articles were included into this meta-analysis. The study flow diagram of the included studies is shown in Figure 1.

The six included RCTs were published from 2013 to 2021, involving data from 1280 participants. Among these people, 567 patients had HIV infections. Of these included studies, the intervention group had rifampin dosages ranging from 13 mg/kg/day intravenously to 35 mg/kg/day orally. The intervention groups in three trials containing 63 participants who were administered high-dose rifampin intravenously, the remained 1,217 patients took rifampin orally. In two studies the intensified regimen involved quinolones, one of them containing levofloxacin at 20 mg/kg/day (11), while another one included moxifloxacin at 400 mg/day or 800 mg/day (10). And in one study from Sean Wasserman, the intervention group treatment consisted of high-dose rifampin plus linezolid, as well as other first-line antituberculosis drugs, one intervention arm applied aspirin at the same time (13). For the method of rifampin concentration detection, in a study by Ruslami (10), rifampin concentration was analyzed by validating high performance liquid chromatography assays (HPLC). The liquid chromatography-tandem mass spectrometry assay was performed to measure the rifampin concentration in two studies by Wasserman and Ding (13, 14). In addition, Cresswell et al. (15) used high-performance liquid chromatography with ultraviolet detection (HPLC-UV) to measure the concentration of rifampin in plasma and CSF. And in the study conducted by Dian S (16), concentration was assessed through the ultraperformance liquid chromatography method (UPLC). The basic characteristics of each study are displayed in Tables 1, 2.

**Table 1:** Details of each enrolled study.

**References**	**Country**	**Study type**	**Number of patient** **(standard/higher)**
Cresswell et al. (15)	Uganda	RCT	61 (21/40)
Heemskerk et al. (11)	Vietnam	RCT	817 (409/408)
Ruslami et al. (10)	Indonesia	RCT	60 (31/29)
Ding et al. (14)	Vietnam	RCT	233 (118/115)
Dian et al. (16)	Indonesia	RCT	60 (20/40)
Wasserman et al. (13)	South Africa	RCT	49 (19/30)

*RCT, randomized controlled trial*.

**Table 2:** Baseline characteristics of patients in each enrolled trial.

**References**	**HIV**	**Median CD4 count** **(cell/μl)**	**Corticosteroids**	**Follow-up period**	**Standard rifampin** **dosage (mg/kg/day)**	**Standard treatment regimen**	**High rifampin dosage** **(mg/kg/day)**	**Intervention time**	**Intensified regimen**	**Outcomes**
Cresswell et al. (15)	56	50	All	6 m	10	HRZE	Arm 1: IV-20 Arm 2: PO-35	8 weeks	HRZE	①②③
Heemskerk et al. (11)	349	38	All	9 m	10	HRZE	PO-15	8 weeks	HRZELfx	②③
Ruslami et al. (10)	7	NA	All	6 m	10	HRZEMfx	IV-13	14 days	HRZEMfx	①②③
Ding et al. (14)	100	NA	All	9 m	10	HRZE	PO-15	8 weeks	HRZELfx	①
Dian et al. (16)	6	NA	All	6 m	10	HRZE	Arm 1: PO-20 Arm 2: PO-30	30 days	HRZE	①②③
Wasserman et al. (13)	49	113	All	6 m	10	HRZE	Arm 1: IV-20 Arm 2: PO-35	56 days	Arm 1: HRZELzd, Arm 2: HRZELzd, and Asp	①

*HIV, human immunodeficiency virus; m, months; H, isoniazide; R, rifampin; E, ethambutol; Z, pyrazinamide; Mfx, moxifloxacin; Lfx, levofloxacin; Lzd, linezolid; Asp, aspirin; IV, intravenous; PO, per os. ① Pharmacokinetic parameters; ② survival; ③ adverse events*.

### Risk of Bias Assessment

**Figure 2:**
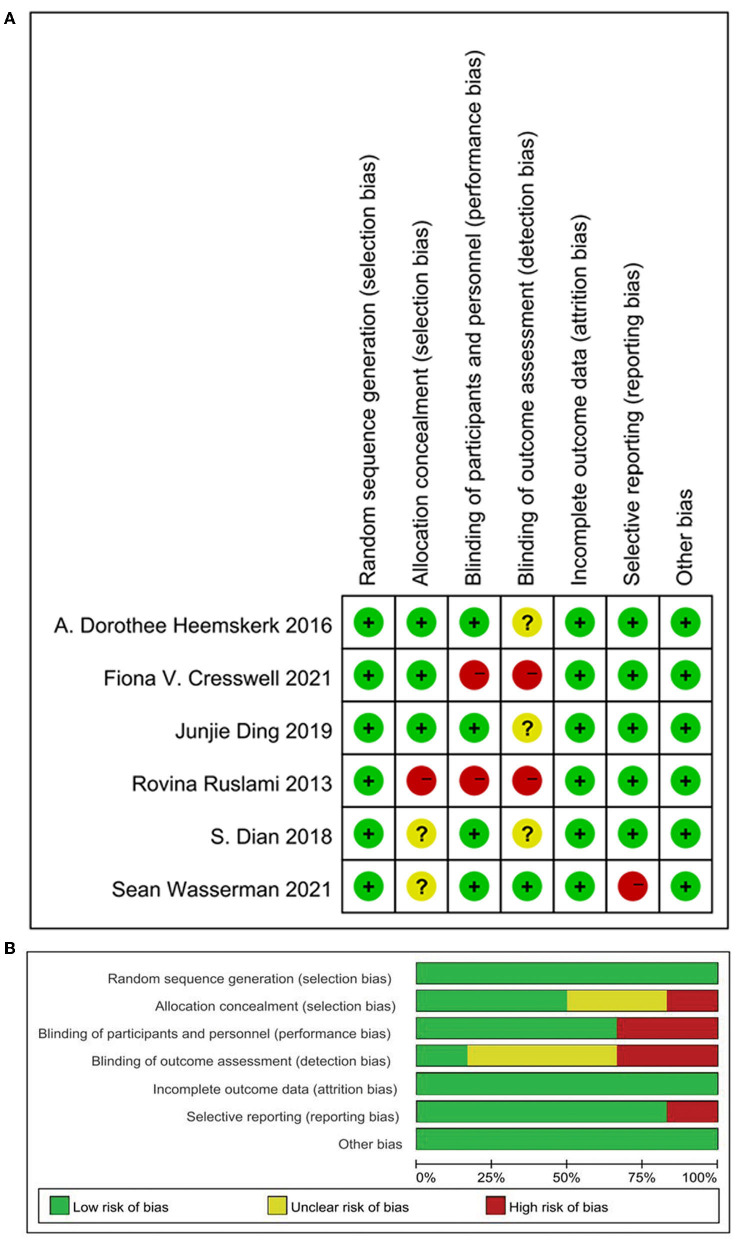
Quality assessment of the studies. **(A)** Risk of bias summary: Review authors' judgements about each risk of bias item for each included study; **(B)** risk of bias graph: Review authors' judgements about each risk of bias item presented as percentages across all included studies.

The Cochrane Risk of Bias Tool was applied to assess the risk of bias of the included studies. Two studies could not be blinded because intravenous and oral administration of rifampin was compared (10, 15), so these studies were found to have a high risk of performance bias and detection bias. The study conducted by Wasserman and colleagues also had two intervention groups, which received either high-dose oral rifampin at 35 mg/kg/day or intravenously at 20 mg/kg/day. It had a low risk of performance and detection bias as the outcome measure was an objective PK endpoint; we found that lack of participant blinding could not have significantly affected the outcome (13). In summary, all the included studies had a low risk of selection, attribution, and other bias. The study conducted by Ruslami (10) had a high risk of selection, performance, and detection bias as explained above. The trials reported by Wasserman and Dian (13, 16) had an unclear risk of allocation concealment due to missing data. The studies conducted by Heemskerk et al. (11, 14, 16) had an unclear risk of detection bias as they did not clearly illustrate the method used in personnel and outcome assessments. Details on the risk of bias assessment are shown in Figures 2A,B.

### Primary Outcomes

#### Plasma Maximum Concentration (C_max_)

**Figure 3:**
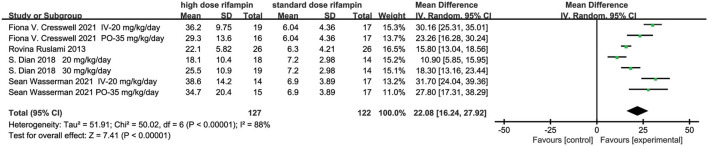
Forest plot result of the maximum concentration of rifampin in plasma.

Four eligible studies reported the plasma C_max_ of rifampin (10, 13, 15, 16). The random effects model meta-analysis showed that there were significant differences in plasma rifampin C_max_ in the overall effects [MD = 22.08, 95%CI (16.24, 27.92), *p* < 0.00001; Figure 3]. Remarkable heterogeneity was observed through the heterogeneity test, with *I*^2^ = 88%. Subgroup analyses were conducted to explore the sources of heterogeneity, containing rifampin application methods, higher dose rifampin treatment duration, rifampin dosage, and whether the intensified regimen used drugs besides HZE (Supplementary Figure 1). As we can see, one of the subgroup's heterogeneity was significantly declined when the studies were divided into two subgroups on the basis of intervention treatment duration and dosage of rifampin. The result suggested that the dose of the rifampin given and the length of administration time may be the major source of heterogeneity.

#### Area Under the Time Concentration Curve Between 0 and 24 h (AUC_0–24_)

**Figure 4:**
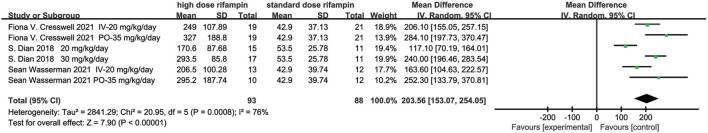
Forest plot result of the AUC0-24 of rifampin.

Three articles evaluated the rifampin AUC_0−24_ (13, 15, 16). The meta-analysis results showed there were statistical differences in rifampin AUC_0−24_ between the higher dose rifampin group and standard group [MD 203.56, 95%CI (153.07, 254.05), *p* < 0.00001; Figure 4]. The heterogeneity was 76%, which was assessed by I^2^. Through the subgroup analysis, the rifampin dose and intervention treatment period were the most probable reason for heterogeneity (Supplementary Figure 2).

#### The CSF Concentration

**Figure 5:**
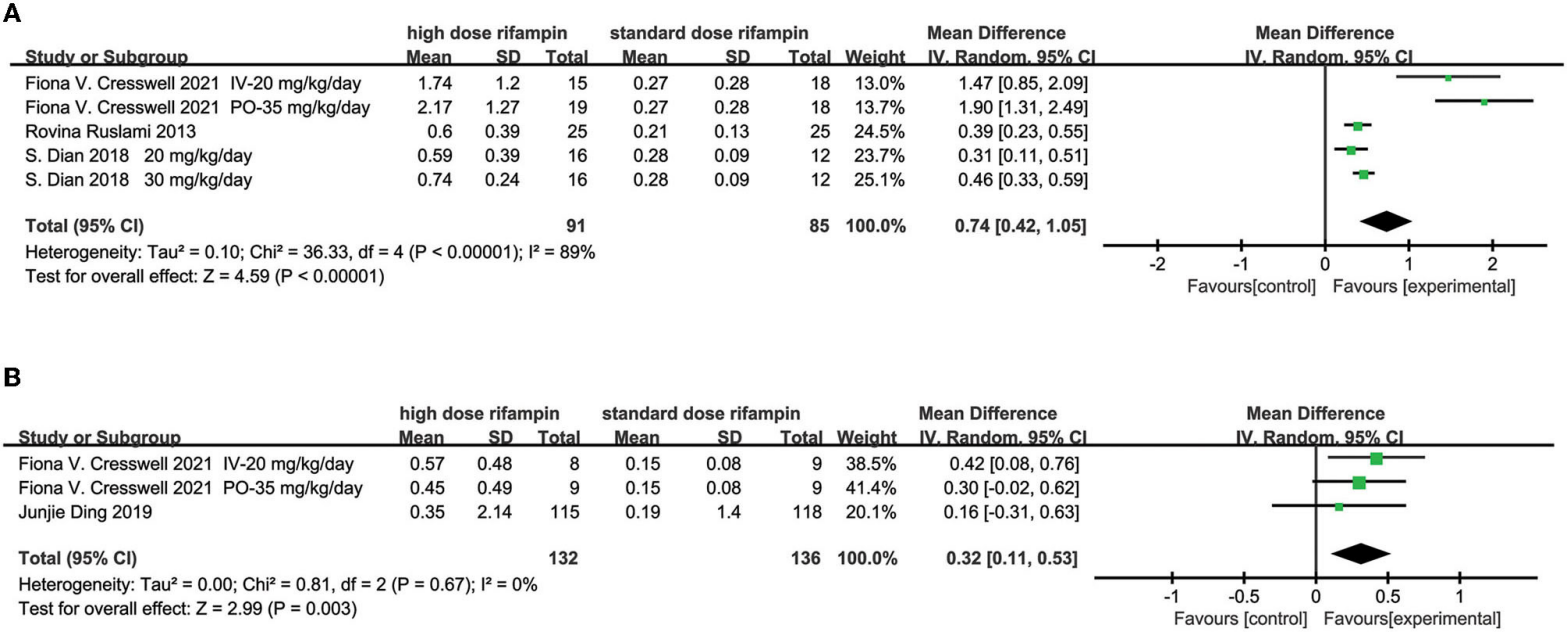
Forest plot results of the concentration of rifampin in CSF. **(A)** The result in the first 3 days; **(B)** the result at day 14.

Three studies described the CSF concentration in the first 3 days (10, 15, 16) and two articles at day 14 (14, 15). The overall effect size conducted by the random effects model demonstrated a significant difference between the intervention group and control group in the first 3 days [MD = 0.74, 95%CI (0.42, 1.05), *p* < 0.00001; Figure 5A]. The heterogeneity was measured as an I^2^ of 89%. Although we conducted subgroup analysis to investigate the source of heterogeneity including the rifampin dosage and the intervention time, unfortunately, we could not reasonably explain the origin of the heterogeneity (Supplementary Figure 3). At day 14, the meta-analysis results showed that there was a statistical difference in the CSF concentration [MD = 0.32, 95%CI (0.11, 0.53), *p* = 0.003; Figure 5B].

### Secondary Outcomes

#### Mortality

**Figure 6:**
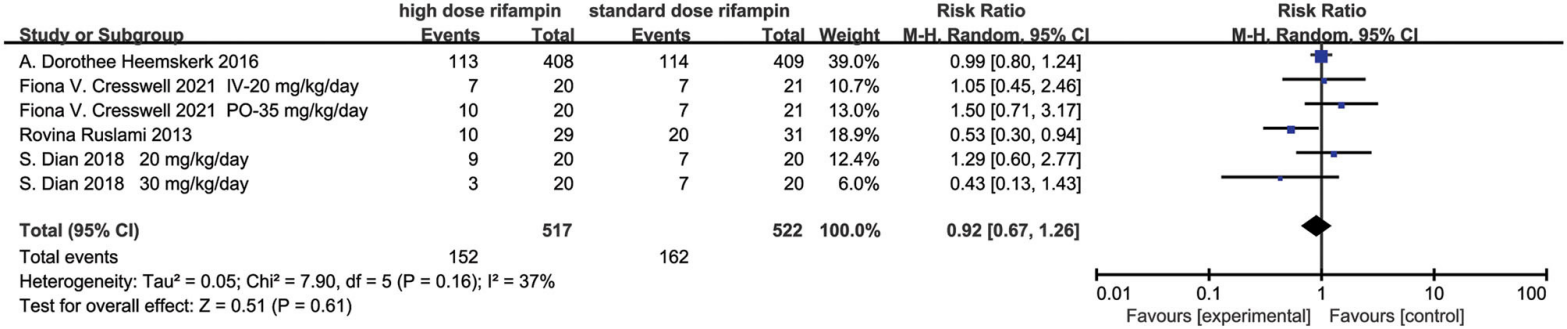
Forest plot result of mortality.

Four studies included in this meta-analysis evaluated the mortality in patients diagnosed with tuberculous meningitis given intensified or standard therapy within 9 months after randomization (10, 11, 15, 16). The pooled results showed that there was no statistical difference under a random effects model [RR = 0.92, 95%CI (0.67, 1.26), *p* = 0.61; Figure 6]. The heterogeneity was described as an *I*^2^ of 37%, therefore we consider that there was no heterogeneity in this analysis statistically.

#### Adverse Events

Four studies recorded adverse events (AEs) (10, 11, 15, 16), we mainly discussed the hepatotoxicity, hypersensitivity, neurological events, anemia, and cardiotoxicity. Statistical heterogeneity was not observed, which was measured by *I*^2^.

##### Hepatotoxicity

**Figure 7:**
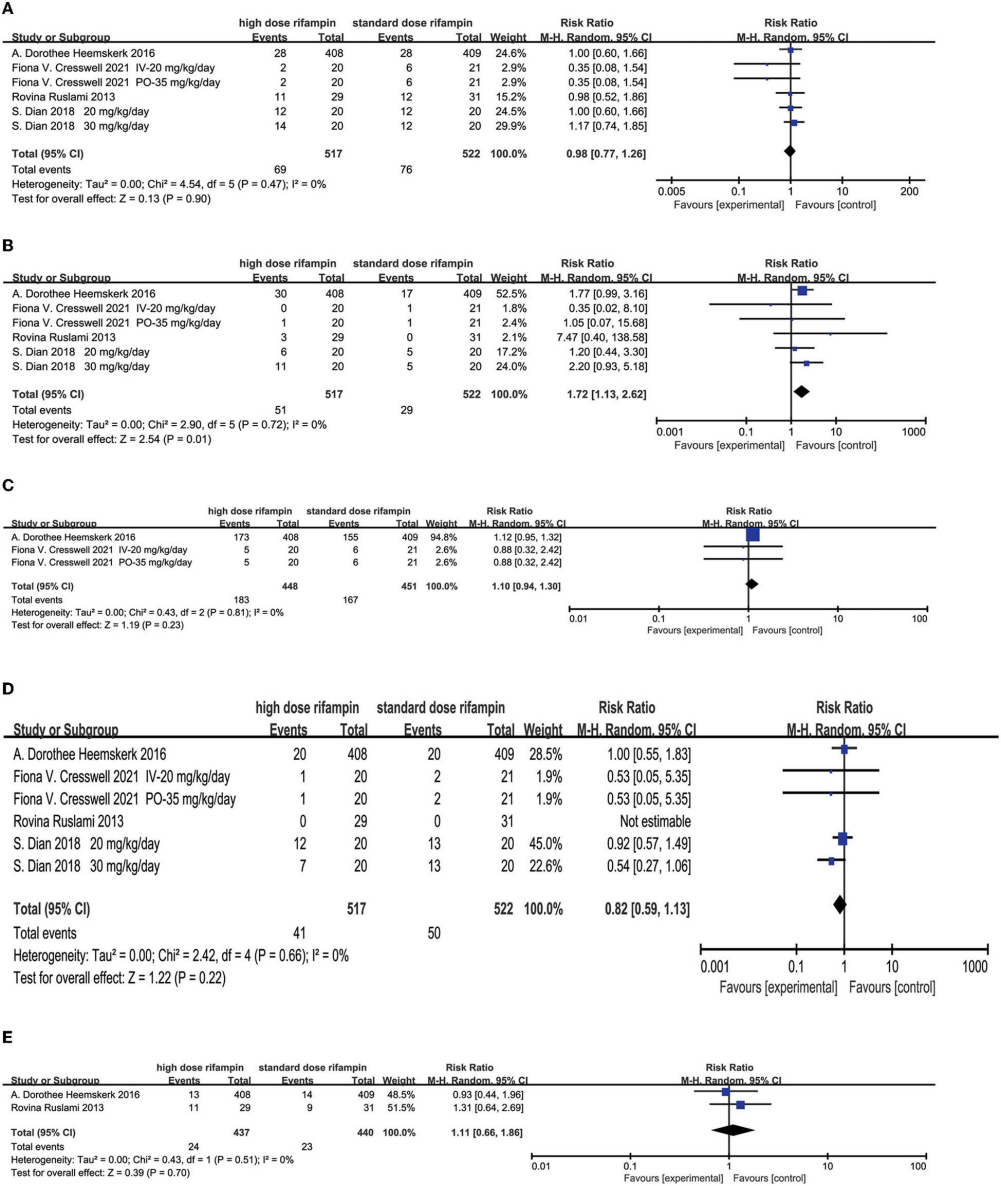
Forest plot results of the occurrence of adverse events. **(A)** The result of liver injury; **(B)** the result of hypersensitivity; **(C)** the result of neurological events; **(D)** the result of myelosuppression; **(E)** the result of cardiologic events.

The overall effect by the random effects model demonstrated that hepatotoxicity had no significant difference among the two groups [RR = 0.98, 95%CI (0.77, 1.26), *p* = 0.90; Figure 7A].

##### Hypersensitivity

The occurrence of developing an allergic reaction was found with statistical significance in the high-dose rifampin arm compared with the standard dose arm [RR = 1.72, 95%CI (1.13, 2.62), *p* = 0.01; Figure 7B].

##### Neurological Events

Two studies (11, 15) reported neurological events mainly containing seizures, neuropathy, altered mental status, cerebrovascular accident, headache, cranial nerve palsies, vision impairment, hearing loss, paralysis, and so on. The pooled results after a random effects model showed there was no significant difference in the incidence of neurological disorders [RR = 1.10, 95%CI (0.94, 1.30), *p* = 0.23; Figure 7C].

##### Myelosuppression

Four articles (10, 11, 15, 16) described the proportion of patients presenting myelosuppression. The overall effect using the random effects model showed that no significant difference was found in the appearance of myelosuppression among the intervention group and standard group [RR = 0.82, 95%CI (0.59, 1.13), *p* = 0.22; Figure 7D].

##### Cardiologic Events

Two studies conducted by Heemskerk (11) and Ruslami (10) reported the outcome of developed cardiotoxicity. The meta-analysis results showed that there were no significant differences between the two groups [RR = 1.11, 95%CI (0.66, 1.86), *p* = 0.70; Figure 7E].

## Discussion

The mortality and disability rates of TBM are still high despite sufficient antituberculosis medicated therapy and the use of adjunctive corticosteroids, so it is very necessary to find new ways to improve the treatment outcome of TBM. Due to the long time it takes to explore new drugs for TB, the best way is to make full use of existing drugs. In recent years, a higher dose of rifampin for TBM has been assessed in several articles. Thus, in this systematic review and meta-analysis, we evaluated the pharmacokinetic parameters of rifampin in CSF and plasma, as well as the safety and survival benefit of the antituberculosis therapy regimen including a higher dose of rifampin. We found that increasing the dosage of rifampin could obviously increase the rifampin concentration in CSF and plasma, however, without significantly improving survival rates compared with the standard regimen. For adverse events, no significant difference was found in the safety between the two groups besides the occurrence of hypersensitivity.

Rifampin as a valuable agent in managing tuberculosis, displays early bactericidal activity, leading to a rapidly decrease in bacillary load in a few days after the start of antituberculosis therapy and preventing post-treatment relapse (17, 18). Lower rifampin in body fluid may affect the treatment efficiency. For instance, a retrospective study by Tongeren et al. in 2013 demonstrated that low drug levels were commonly observed in the population who had treatment failure and acquired drug resistance (19). Recently, a study conducted by Sekaggya-Wiltshire and colleagues enrolled 268 pulmonary tuberculosis patients co-infected with HIV, the results found that low concentration of isoniazid and rifampin had a relationship with delayed sputum culture conversion, which may affect the spread of tuberculosis (20). However, a systematic review and meta-analysis in 2016 found that a majority of people had a sub-standard antituberculosis drug concentration compared with the accepted normal criteria (21). The results of this meta-analysis showed that rifampin exhibits a dose-dependent increase in serum concentration, the finding is consistent with many other studies in pulmonary tuberculosis. A multiple dose-ranging study in culture-positive pulmonary tuberculosis patients performed by Boeree et al. (22) suggested a noteworthy increase in rifampin C_max_ along with the dose increase of rifampin. When administering the dose at 35 mg/kg/day for 2 weeks, the average AUC_0−24_ was approximately 10 times higher than the exposure of rifampin at 10 mg/kg/day, from 26.3 to 235.4 h.mg/L. The same results were observed in a trial enrolling patients diagnosed with pulmonary TB who were given a regimen containing a higher dose of oral rifampin at 15 and 20 mg/kg daily for a longer period of 2 months, which resulted in a more than proportional increase in the level of rifampin exposure in plasma (23). There are several possible explanations for the results: (1) The dominant metabolite of rifampin is desacetyl-rifampin, both rifampin and desacetyl-rifampin are excreted through bile. As the dosage of rifampin increased, the biliary excretion may reach saturation point (6, 24–26). (2) Rifampin is a critical inducer of many metabolizing enzymes, having an obvious influence on the expression of cytochrome P450 (CYP) 3A4 in the liver. When rifampin is administered repeatedly, it will induce its own metabolizing hepatic enzymes resulting in the decrease of plasma concentration, which would lead to a reduction of bioavailability for rifampin (27, 28). Therefore, if rifampin is delivered at a higher than standard dose, the ability of the liver to metabolize rifampin may approach saturation, furthermore, leading to the increase of rifampin plasma concentration (24, 27). (3) As rifampin's binding rate of plasma protein is fairly high (6), the free plasma rifampin possibly increased as the rifampin dosage enhanced.

For CNS infections, the appropriate management requires antimicrobial medications to penetrate the blood-brain barrier and be able to achieve a satisfactory CSF concentration that meets the pharmacodynamics criteria, at least above the MIC (15, 29), which is defined as the lowest concentration of an antimicrobial that will inhibit the visible growth of a microorganism after overnight incubation (6, 7, 15, 30). However, the ability of rifampin to penetrate to the central nervous system with the present WHO recommended dose is poor, the CSF concentration barely exceeds the MIC of *Mycobacterium tuberculosis* (6, 7, 15). According to this meta-analysis, the increased rifampin dose resulted in a dramatic rifampin concentration increase in CSF in the first 3 days, as well as at 14 days after initiation of treatment. The steady state created by the auto-induction of rifampin clearance was nearly complete which led to reduced plasma levels (31). The result may be attributed to the increased circulating plasma concentrations. Amanda et al. believed that highly protein-bound drugs are more difficult to transport into the CNS as fewer free parts were available. Therefore, this kind of drug's CSF concentration mainly depends on circulating plasma concentration (29). Furthermore, a model-based analysis on the basis of three clinical trials relating to the comparison of a higher dose rifampin regimen with the standard regimen came to the same conclusion, that the CSF concentration was closely connected with plasma concentration (31). Thus, in order to reach an effective CSF therapy concentration, increasing the rifampin dose may be a considerable idea.

Drug concentration can be the most important predictor of patient outcomes (32). As we mentioned above, several studies have been made to illustrate the relationship between drug concentration and pulmonary tuberculosis treatment outcomes, in that a lower and suboptimal drug concentration was associated with delayed sputum culture conversion, higher relapse rates, poor treatment outcome, and developed drug resistance (19, 20, 33, 34). Therefore, a number of clinical trials have examined whether increased rifampin dose could improve the therapeutic effect. For example, the study by Boeree et al. (22) described above demonstrated that the rifampin dose of 30 and 35 mg/kg/day for 2 weeks led to the highest 14-day early bactericidal activity as measured by a more obvious fall in counts of colony-forming units (CFU) and a more rapid increase in time to positivity (TTP), moreover, this dose was safe and well-tolerated. On the foundation of Boeree's study, Aarnoutse et al. (23) evaluated a higher dose rifampin of 15 or 20 mg/kg/d for a long period of 2 months in pulmonary tuberculosis, although there was a more than proportional increase in the level of exposure to rifampin in plasma, the results showed that there was no difference between a variable dose of rifampin in the time to culture conversion. The author suggested that the dominant reason for this result was the fact that the rifampin dose and exposure were not high enough to elicit clinical effects. Another study conducted by Velásquez et al. (35) also assessed the efficacy and safety of rifampin at 15 or 20 mg/kg/d during an 8-week intensive phase in patients with pulmonary tuberculosis, the research findings showed higher dose and exposure resulted in an increase in the rate of sputum culture sterilization and did not increase the risk of toxicity, but the 8-week culture conversion and the frequency of treatment failure and relapse had no difference between arms. The theoretical basis of using a higher dose of rifampin is the concentration and effect relationship, the dose of rifampin may have a dominant impact on rifampin's effect. An *in vitro* pharmacokinetic-pharmacodynamic model of tuberculosis demonstrated that even with a focus on microbial killing or resistance suppression, the effect of rifampin was concentration-dependent (17). However, the relationship between drug concentration and clinical microbial response was non-linear and complex; in the concentration-response curve, the positive or better outcomes may show when the concentration goes beyond a certain point or on the steep part of the curve (23, 32). Rifampin exhibits an increase in bactericidal activity with an increased dose, a model-based clinical trial by Svensson (36) simulated early bactericidal activity of 45 and 50 mg/kg rifampin for patients, and predicted a further increase in early bactericidal activity. Based on the above, more studies are needed to verify whether a much higher than standard dose of rifampin could improve the culture conversion rate, cure rate, and death rate.

Unfortunately, despite remarkable increases of plasma and CSF rifampin concentration, this meta-analysis pool results did not show the survival benefit of TBM patients when they took a regimen including a higher dose of rifampin. The results of this meta-analysis are inconsistent with the previous model-based meta-analysis conducted by Svensson et al. (31), which reached a conclusion that a higher dose of rifampin improves survival in TBM patients. We thought that the meta-analysis by Svensson had limited studies and sample sizes, only three clinical trials including 148 patients (133 patients had pharmacokinetic parameters among them), which may affect the accuracy of the results. Moreover, in our meta-analysis, almost half of the patients in the eligible studies had HIV co-infection, and as rifampin can induce many drug-metabolizing enzymes, it has a huge impact on the pharmacokinetics of many drugs, including antiretroviral therapy drugs, and can lead to decreased plasma concentration of antiretroviral drugs (28), which may contribute to the high mortality. Furthermore, we suspected that under these doses of rifampin, the levels of intracerebral drug concentrations achieved were not sufficient to enhance bacterial killing. The study in pulmonary tuberculosis demonstrated that a rifampin dose of 35 mg/kg/day was accepted and safe, the increase in rifampin exposure did not show an apparent ceiling effect (22). Perhaps a much higher dose of rifampin will lead to a better outcome. More research is needed to explore this conjecture. In general, valid comprehensive management may improve the treatment outcome of TBM, including a more sensitive and accurate diagnostic method, optimizing the therapy regimen plus favorable management of complications (4).

There are encouraging signs that although the rifampin level increased, the toxicity almost did not increase, except for the occurrence of hypersensitivity (*p* = 0.01). In accordance with the present results, previous studies have demonstrated that no significant difference in terms of safety between a standard dose rifampin regimen and a higher dose rifampin regimen in pulmonary tuberculosis (35, 37–39). Hepatotoxicity is the most common adverse reaction during antituberculosis chemotherapy, which can lead to liver failure and eventually death. In addition, HIV infection is a major risk factor for hepatotoxicity (40). It is encouraging that in our meta-analysis, the occurrence of liver injury was not statistically different between the two groups.

There are several limitations of this study. First, the administered dose of rifampin, duration of treatment, drug delivery method, and the intensified regimen among eligible studies were different. In some analyses, there was obvious heterogeneity. Second, in this meta-analysis, the most included studies were phase II clinical trials, and due to limited sample size, the analysis was not powered to draw a conclusion of clinical outcome. Third, for the small number of RCTs, the power of some analyses was limited and may affect the accuracy of the results. Fourth, in consideration of the compliance and comfort of the experimental participants, most studies performed a single lumbar puncture, which may lead the conclusion to lack accuracy and persuasion.

## Conclusion

In conclusion, this meta-analysis suggested that a higher dose of rifampin could significantly increase the pharmacokinetic parameters containing plasma and CSF concentration and the plasma AUC_0−24_, without a remarkable increase of adverse reactions. This phenomenon may indicate the idea that the crucial drug in the antituberculosis regimen was used in a low dose, more studies are required to shed light on this question. However, we found that there was no improvement in the treatment outcome. In terms of efficacy, more work will need to be done, especially large sample size phase III studies to determine the effect of an intensified regimen including a higher dose of rifampin.

## Data Availability Statement

The original contributions presented in the study are included in the article/Supplementary Material, further inquiries can be directed to the corresponding author/s.

## Author Contributions

MZ and MW completed the study design, extracted and analyzed the data, and wrote the original draft. J-QH reviewed the final draft. All authors approved the final version.

## Funding

This work was supported by the National Natural Science Foundation of China (Grant No. 81870015).

## Conflict of Interest

The authors declare that the research was conducted in the absence of any commercial or financial relationships that could be construed as a potential conflict of interest.

## Publisher's Note

All claims expressed in this article are solely those of the authors and do not necessarily represent those of their affiliated organizations, or those of the publisher, the editors and the reviewers. Any product that may be evaluated in this article, or claim that may be made by its manufacturer, is not guaranteed or endorsed by the publisher.
