# Supplementary material for: Intensified Antituberculosis Therapy Regimen Containing Higher Dose Rifampin for Tuberculous Meningitis: A Systematic Review and Meta-Analysis
Source: Front Med (Lausanne). 2022 Feb 25;9:822201. doi: 10.3389/fmed.2022.822201 (PMC8916538; doi:10.3389/fmed.2022.822201)
Supplement: Supplementary file 1 [file Data_Sheet_1.PDF]

Supplementary Material

Supplementary Figure 1A.

Study or Subgroup	Experimental Mean	Experimental SD	Experimental Total	Control Mean	Control SD	Control Total	Weight	Mean Difference IV, Random, 95% CI
**2.1.1 Intravenous**								
Fiona V. Cresswell 2021 IV-20 mg/kg/day	36.2	9.75	19	6.04	4.36	17	15.3%	30.16 [25.31, 35.01]
Rovina Ruslami 2013	22.1	5.82	26	6.3	4.21	26	16.5%	15.80 [13.04, 18.56]
Sean Wasserman 2021 IV-20 mg/kg/day	38.6	14.2	14	6.9	3.89	17	13.2%	31.70 [24.04, 39.36]
**Subtotal (95% CI)**			**59**			**60**	**45.0%**	**25.58 [14.14, 37.02]**
Heterogeneity: Tau² = 94.74; Chi² = 34.57, df = 2 (P < 0.00001); I² = 94%								
Test for overall effect: Z = 4.38 (P < 0.0001)								
**2.1.2 Oral**								
Fiona V. Cresswell 2021 PO-35 mg/kg/day	29.3	13.6	16	6.04	4.36	17	13.7%	23.26 [16.28, 30.24]
S. Dian 2018 20 mg/kg/day	18.1	10.4	18	7.2	2.98	14	15.2%	10.90 [5.85, 15.95]
S. Dian 2018 30 mg/kg/day	25.5	10.9	19	7.2	2.98	14	15.1%	18.30 [13.16, 23.44]
Sean Wasserman 2021 PO-35 mg/kg/day	34.7	20.4	15	6.9	3.89	17	11.0%	27.80 [17.31, 38.29]
**Subtotal (95% CI)**			**68**			**62**	**55.0%**	**19.19 [12.45, 25.93]**
Heterogeneity: Tau² = 35.01; Chi² = 12.96, df = 3 (P = 0.005); I² = 77%								
Test for overall effect: Z = 5.58 (P < 0.00001)								
**Total (95% CI)**			**127**			**122**	**100.0%**	**22.08 [16.24, 27.92]**
Heterogeneity: Tau² = 51.91; Chi² = 50.02, df = 6 (P < 0.00001); I² = 88%								
Test for overall effect: Z = 7.41 (P < 0.00001)								
Test for subgroup differences: Chi² = 0.89, df = 1 (P = 0.35), I² = 0%								

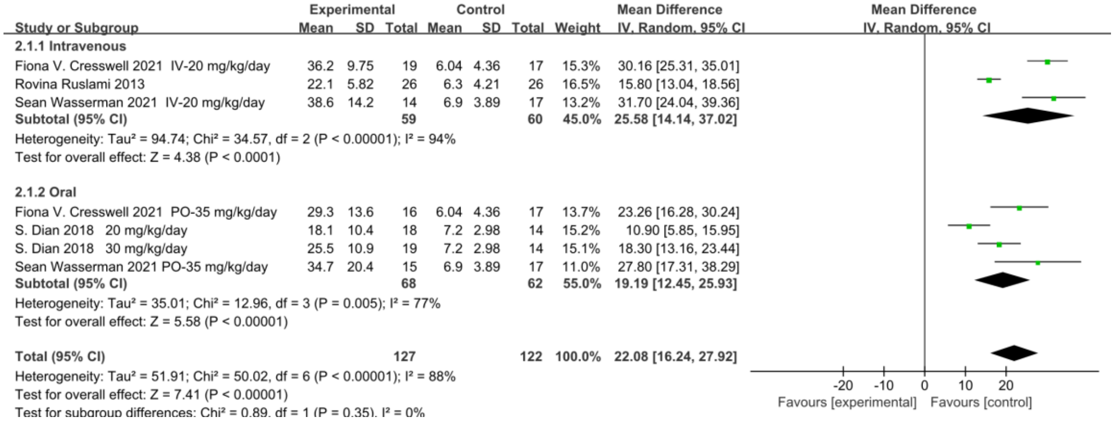

Supplementary Figure 1B.

Study or Subgroup	Experimental Mean	Experimental SD	Experimental Total	Control Mean	Control SD	Control Total	Weight	Mean Difference IV, Random, 95% CI
**2.3.1 HRZE regimen**								
Fiona V. Cresswell 2021 IV-20 mg/kg/day	36.2	9.75	19	6.04	4.36	17	15.3%	30.16 [25.31, 35.01]
Fiona V. Cresswell 2021 PO-35 mg/kg/day	29.3	13.6	16	6.04	4.36	17	13.7%	23.26 [16.28, 30.24]
S. Dian 2018 20 mg/kg/day	18.1	10.4	18	7.2	2.98	14	15.2%	10.90 [5.85, 15.95]
S. Dian 2018 30 mg/kg/day	25.5	10.9	19	7.2	2.98	14	15.1%	18.30 [13.16, 23.44]
**Subtotal (95% CI)**			**72**			**62**	**59.3%**	**20.62 [12.01, 29.24]**
Heterogeneity: Tau² = 69.33; Chi² = 30.42, df = 3 (P < 0.00001); I² = 90%								
Test for overall effect: Z = 4.69 (P < 0.00001)								
**2.3.2 HRZE alongside other drugs**								
Rovina Ruslami 2013	22.1	5.82	26	6.3	4.21	26	16.5%	15.80 [13.04, 18.56]
Sean Wasserman 2021 IV-20 mg/kg/day	38.6	14.2	14	6.9	3.89	17	13.2%	31.70 [24.04, 39.36]
Sean Wasserman 2021 PO-35 mg/kg/day	34.7	20.4	15	6.9	3.89	17	11.0%	27.80 [17.31, 38.29]
**Subtotal (95% CI)**			**55**			**60**	**40.7%**	**24.57 [12.92, 36.21]**
Heterogeneity: Tau² = 91.71; Chi² = 18.04, df = 2 (P = 0.0001); I² = 89%								
Test for overall effect: Z = 4.13 (P < 0.0001)								
**Total (95% CI)**			**127**			**122**	**100.0%**	**22.08 [16.24, 27.92]**
Heterogeneity: Tau² = 51.91; Chi² = 50.02, df = 6 (P < 0.00001); I² = 88%								
Test for overall effect: Z = 7.41 (P < 0.00001)								
Test for subgroup differences: Chi² = 0.28, df = 1 (P = 0.59), I² = 0%								

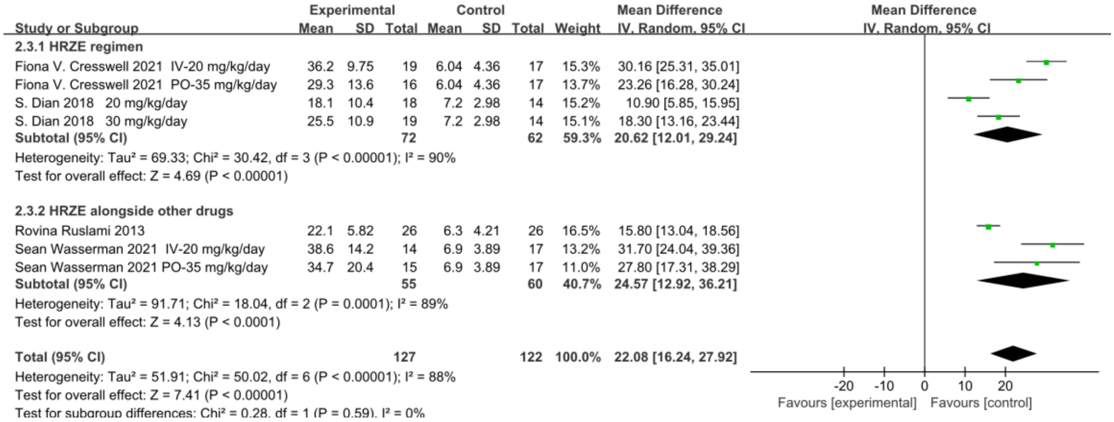

Supplementary Figure 1C.

Study or Subgroup	Experimental Mean	Experimental SD	Experimental Total	Control Mean	Control SD	Control Total	Weight	Mean Difference IV, Random, 95% CI
**2.4.1 less than or equal to 20mg/kg/day**								
Fiona V. Cresswell 2021 IV-20 mg/kg/day	36.2	9.75	19	6.04	4.36	17	15.3%	30.16 [25.31, 35.01]
Rovina Ruslami 2013	22.1	5.82	26	6.3	4.21	26	16.5%	15.80 [13.04, 18.56]
S. Dian 2018 20 mg/kg/day	18.1	10.4	18	7.2	2.98	14	15.2%	10.90 [5.85, 15.95]
Sean Wasserman 2021 IV-20 mg/kg/day	38.6	14.2	14	6.9	3.89	17	13.2%	31.70 [24.04, 39.36]
**Subtotal (95% CI)**			**77**			**74**	**60.1%**	**21.84 [12.68, 31.01]**
Heterogeneity: Tau² = 80.18; Chi² = 45.89, df = 3 (P < 0.00001); I² = 93%								
Test for overall effect: Z = 4.67 (P < 0.00001)								
**2.4.2 more than 20mg/kg/day**								
Fiona V. Cresswell 2021 PO-35 mg/kg/day	29.3	13.6	16	6.04	4.36	17	13.7%	23.26 [16.28, 30.24]
S. Dian 2018 30 mg/kg/day	25.5	10.9	19	7.2	2.98	14	15.1%	18.30 [13.16, 23.44]
Sean Wasserman 2021 PO-35 mg/kg/day	34.7	20.4	15	6.9	3.89	17	11.0%	27.80 [17.31, 38.29]
**Subtotal (95% CI)**			**50**			**48**	**39.9%**	**21.75 [16.67, 26.83]**
Heterogeneity: Tau² = 7.21; Chi² = 3.07, df = 2 (P = 0.22); I² = 35%								
Test for overall effect: Z = 8.40 (P < 0.00001)								
**Total (95% CI)**			**127**			**122**	**100.0%**	**22.08 [16.24, 27.92]**
Heterogeneity: Tau² = 51.91; Chi² = 50.02, df = 6 (P < 0.00001); I² = 88%								
Test for overall effect: Z = 7.41 (P < 0.00001)								
Test for subgroup differences: Chi² = 0.00, df = 1 (P = 0.99), I² = 0%								

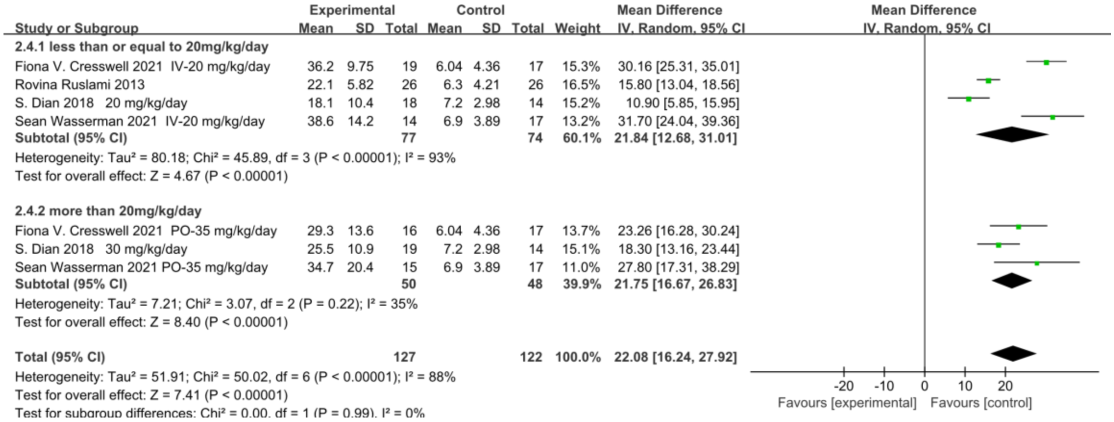

Supplementary Figure 1D.

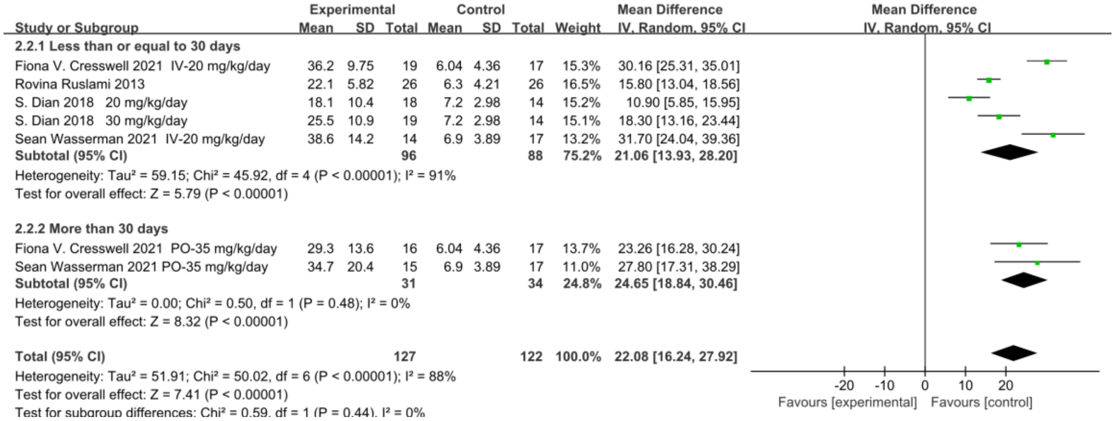

Supplementary Figure 1.The subgroup analysis of the maximum concentration of rifampin in plasm. (A) The subgroup was divided by drug delivery method;(B) The subgroup was divided according to therapeutic regimen;(C) The subgroup was stratified through the dose of medicine;(D) The subgroup was divided according to the treatment duration

Supplementary Figure 2A.

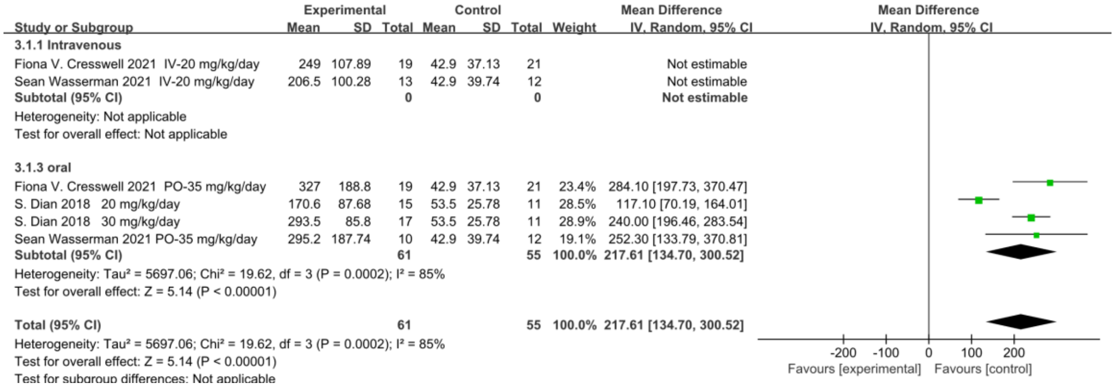

Supplementary Figure 2B

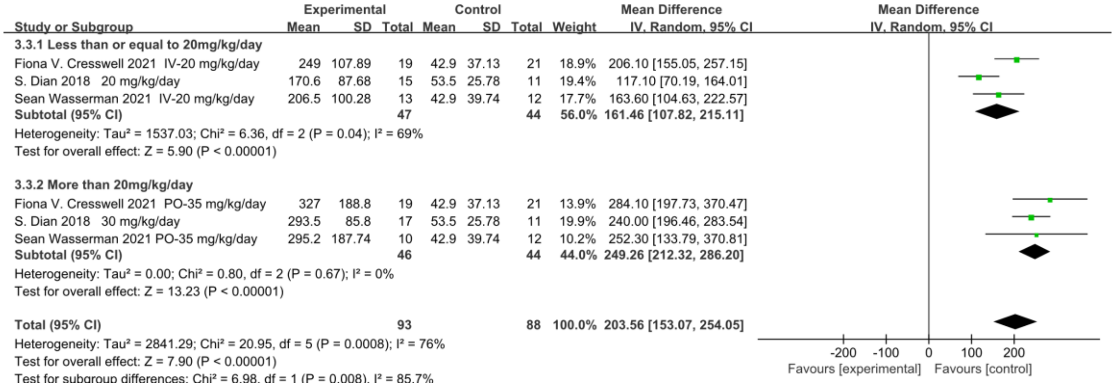

Supplementary Figure 2C.

Study or Subgroup	Experimental Mean	SD	Total	Control Mean	SD	Total	Weight	Mean Difference IV, Random, 95% CI
**3.2.1 Less than or equal to 30 days**								
Fiona V. Cresswell 2021 IV-20 mg/kg/day	249	107.89	19	42.9	37.13	21	18.9%	206.10 [155.05, 257.15]
S. Dian 2018 20 mg/kg/day	170.6	87.68	15	53.5	25.78	11	19.4%	117.10 [70.19, 164.01]
S. Dian 2018 30 mg/kg/day	293.5	85.8	17	53.5	25.78	11	19.9%	240.00 [196.46, 283.54]
Sean Wasserman 2021 IV-20 mg/kg/day	206.5	100.28	13	42.9	39.74	12	17.7%	163.60 [104.63, 222.57]
**Subtotal (95% CI)**			**64**			**55**	**75.9%**	**182.32 [126.14, 238.50]**
Heterogeneity: Tau² = 2630.87; Chi² = 15.34, df = 3 (P = 0.002); I² = 80%								
Test for overall effect: Z = 6.36 (P < 0.00001)								
**3.2.2 More than 30 days**								
Fiona V. Cresswell 2021 PO-35 mg/kg/day	327	188.8	19	42.9	37.13	21	13.9%	284.10 [197.73, 370.47]
Sean Wasserman 2021 PO-35 mg/kg/day	295.2	187.74	10	42.9	39.74	12	10.2%	252.30 [133.79, 370.81]
**Subtotal (95% CI)**			**29**			**33**	**24.1%**	**273.07 [203.27, 342.87]**
Heterogeneity: Tau² = 0.00; Chi² = 0.18, df = 1 (P = 0.67); I² = 0%								
Test for overall effect: Z = 7.67 (P < 0.00001)								
**Total (95% CI)**			**93**			**88**	**100.0%**	**203.56 [153.07, 254.05]**
Heterogeneity: Tau² = 2841.29; Chi² = 20.95, df = 5 (P = 0.0008); I² = 76%								
Test for overall effect: Z = 7.90 (P < 0.00001)								
Test for subgroup differences: Chi² = 3.94, df = 1 (P = 0.05), I² = 74.6%								

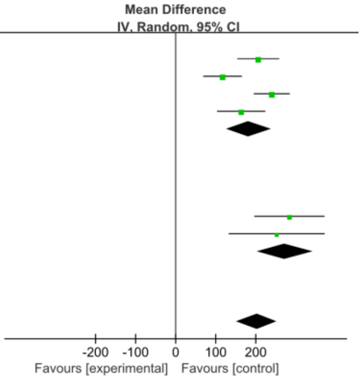

a) **Supplementary Figure 2.** The subgroup analysis of the AUC0-24 of rifampin in plasm. (A) The subgroup was divided by drug delivery method;(B) The subgroup was divided through the dose of medicine;(C) The subgroup was divided according to the high dose rifampin treatment duration.

Supplementary Figure 3A.

Study or Subgroup	Experimental Mean	SD	Total	Control Mean	SD	Total	Weight	Mean Difference IV, Random, 95% CI
**4.1.1 Intravenous**								
Fiona V. Cresswell 2021 IV-20 mg/kg/day	1.74	1.2	15	0.27	0.28	18	13.0%	1.47 [0.85, 2.09]
Rovina Ruslami 2013	0.6	0.39	25	0.21	0.13	25	24.5%	0.39 [0.23, 0.55]
**Subtotal (95% CI)**			**40**			**43**	**37.5%**	**0.89 [-0.17, 1.94]**
Heterogeneity: Tau² = 0.53; Chi² = 10.89, df = 1 (P = 0.0010); I² = 91%								
Test for overall effect: Z = 1.65 (P = 0.10)								
**4.1.2 oral**								
Fiona V. Cresswell 2021 PO-35 mg/kg/day	2.17	1.27	19	0.27	0.28	18	13.7%	1.90 [1.31, 2.49]
S. Dian 2018 20 mg/kg/day	0.59	0.39	16	0.28	0.09	12	23.7%	0.31 [0.11, 0.51]
S. Dian 2018 30 mg/kg/day	0.74	0.24	16	0.28	0.09	12	25.1%	0.46 [0.33, 0.59]
**Subtotal (95% CI)**			**51**			**42**	**62.5%**	**0.76 [0.28, 1.24]**
Heterogeneity: Tau² = 0.15; Chi² = 25.44, df = 2 (P < 0.00001); I² = 92%								
Test for overall effect: Z = 3.12 (P = 0.002)								
**Total (95% CI)**			**91**			**85**	**100.0%**	**0.74 [0.42, 1.05]**
Heterogeneity: Tau² = 0.10; Chi² = 36.33, df = 4 (P < 0.00001); I² = 89%								
Test for overall effect: Z = 4.59 (P < 0.00001)								
Test for subgroup differences: Chi² = 0.05, df = 1 (P = 0.83), I² = 0%								

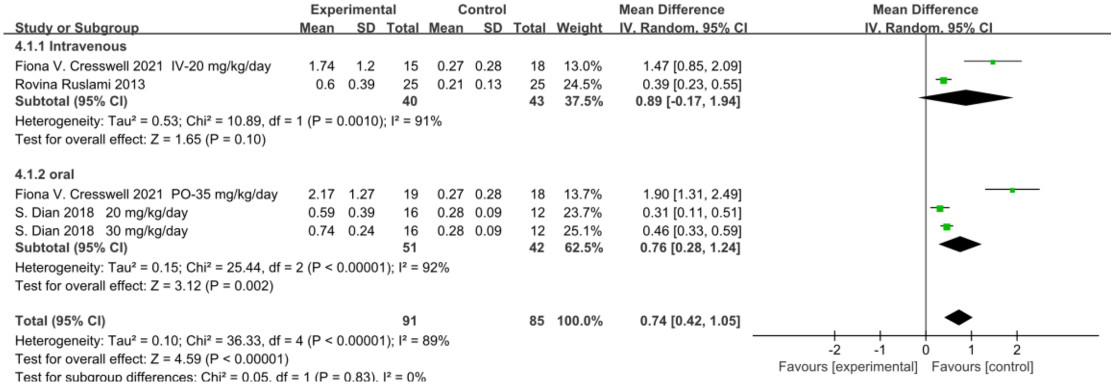

Supplementary Figure 3B.

Study or Subgroup	Experimental Mean	SD	Total	Control Mean	SD	Total	Weight	Mean Difference IV, Random, 95% CI
**4.2.1 Less than or equal to 20mg/kg/day**								
Fiona V. Cresswell 2021 IV-20 mg/kg/day	1.74	1.2	15	0.27	0.28	18	13.0%	1.47 [0.85, 2.09]
Rovina Ruslami 2013	0.6	0.39	25	0.21	0.13	25	24.5%	0.39 [0.23, 0.55]
S. Dian 2018 20 mg/kg/day	0.59	0.39	16	0.28	0.09	12	23.7%	0.31 [0.11, 0.51]
**Subtotal (95% CI)**			**56**			**55**	**61.2%**	**0.57 [0.20, 0.93]**
Heterogeneity: Tau² = 0.08; Chi² = 12.22, df = 2 (P = 0.002); I² = 84%								
Test for overall effect: Z = 3.06 (P = 0.002)								
**4.2.2 More than 20mg/kg/day**								
Fiona V. Cresswell 2021 PO-35 mg/kg/day	2.17	1.27	19	0.27	0.28	18	13.7%	1.90 [1.31, 2.49]
S. Dian 2018 30 mg/kg/day	0.74	0.24	16	0.28	0.09	12	25.1%	0.46 [0.33, 0.59]
**Subtotal (95% CI)**			**35**			**30**	**38.8%**	**1.15 [-0.26, 2.56]**
Heterogeneity: Tau² = 0.99; Chi² = 22.17, df = 1 (P < 0.00001); I² = 95%								
Test for overall effect: Z = 1.60 (P = 0.11)								
**Total (95% CI)**			**91**			**85**	**100.0%**	**0.74 [0.42, 1.05]**
Heterogeneity: Tau² = 0.10; Chi² = 36.33, df = 4 (P < 0.00001); I² = 89%								
Test for overall effect: Z = 4.59 (P < 0.00001)								
Test for subgroup differences: Chi² = 0.61, df = 1 (P = 0.43), I² = 0%								

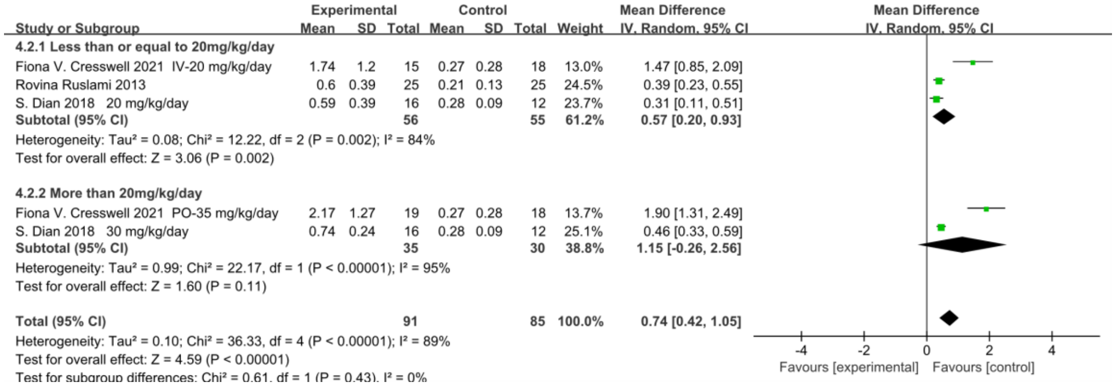

a) **Supplementary Figure 3.** The subgroup analysis of the concentration of rifampin in CSF. (A) The subgroup was divided according to drug application method;(B) The subgroup was divided through the dosage of rifampin.
